# Expression of the methionine sulfoxide reductase lost during evolution extends *Drosophila* lifespan in a methionine-dependent manner

**DOI:** 10.1038/s41598-017-15090-5

**Published:** 2018-01-17

**Authors:** Byung Cheon Lee, Hae Min Lee, Sorah Kim, Andrei S. Avanesov, Aro Lee, Bok-Hwan Chun, Gerd Vorbruggen, Vadim N. Gladyshev

**Affiliations:** 1 College of Life Sciences and Biotechnology, Korea University, Seoul, 02841 South Korea; 2 Division of Genetics, Department of Medicine, Brigham & Women’s Hospital and Harvard Medical School, Boston, MA 02115 USA; 3 Abteilung Molekulare Entwicklungsbiologie, Max-Planck-Institut für biophysikalische Chemie, Göttingen, Germany; 4 Georg-August-Universität Göttingen, GZMB, Abteilung Entwicklungsbiologie, Göttingen, Germany

## Abstract

Accumulation of oxidized amino acids, including methionine, has been implicated in aging. The ability to reduce one of the products of methionine oxidation, free methionine-R-sulfoxide (Met-*R*-SO), is widespread in microorganisms, but during evolution this function, conferred by the enzyme fRMsr, was lost in metazoa. We examined whether restoration of the fRMsr function in an animal can alleviate the consequences of methionine oxidation. Ectopic expression of yeast fRMsr supported the ability of *Drosophila* to catalyze free Met-*R*-SO reduction without affecting fecundity, food consumption, and response to starvation. fRMsr expression also increased resistance to oxidative stress. Moreover, it extended lifespan of flies in a methionine-dependent manner. Thus, expression of an oxidoreductase lost during evolution can enhance metabolic and redox functions and lead to an increase in lifespan in an animal model. More broadly, our study exposes the potential of a combination of genetic and nutritional strategies in lifespan control.

## Introduction

Molecular damage has been widely implicated in the aging process, including the damage caused by reactive oxygen species (ROS)^1–4^. Organisms are exposed to the threat of ROS during aerobic metabolism, which is associated with various reactions involving incompletely reduced species of molecular oxygen^5–7^. ROS may adversely affect biomolecules, such as DNA, lipids, and proteins, and as such have been implicated in the etiology of many disorders as well as in accelerated aging^4,8^. To prevent deleterious accumulation of oxidative damage, organisms use enzymes that participate in the removal of ROS or its consequences, often in cooperation with certain low molecular weight molecules. One such strategy is to repair oxidized biomolecules, restoring their biological functions. Many oxidoreductases have been characterized for their contributions to redox homeostasis and aging such as thioredoxin reductase, methionine sulfoxide reductase (Msr), superoxide dismutase, catalase, and other enzymes^9^.

Several such enzymes rely on the reversible Cys and Met oxidation/reduction, including Msrs which reduces oxidized Met back to Met^10,11^. Together with Cys, Met is the amino acid most susceptible to oxidation by ROS, and its oxidation results in a mixture of methionine-*S*-sulfoxide (Met-*S*-SO) and methionine-*R*-sulfoxide (Met-*R*-SO). One of the Msrs, MsrA, can reduce both protein-based and free Met-*S*-SO, whereas another enzyme, MsrB, can reduce protein-based Met-*R*-SO, but has low efficiency with free Met-*R*-SO^12^. An additional Msr, fRMsr, was also functionally characterized that possesses high efficiency in reducing free Met-*R*-SO, but this enzyme was lost in animals and many plants^13,14^. In this regard, higher orgarisms, such as mammals and insects, are thought to reduce free Met-*R*-SO inefficiently^15^. Like several other antioxidant enzymes, MsrA was implicated in lifespan control in some organisms such as fruit flies and yeast, although not in mouse^16–22^, whereas MsrB and fRMsr are poorly understood with regard to their roles in lifespan control. The observed species-specific regulation of lifespan by Msrs requires further studies. In general, the effects on lifespan observed in invertebrates are more extensive than in mammals.

Interestingly, whereas MsrA expression extended the lifespan of fruit flies^17^, expression of MsrB did not^23^, suggesting that this extension may be dependent on the reduction of free methionine sulfoxide (Met-*S*-SO in the case of MsrA). If so, the inability to reduce free Met-*R*-SO may be the Achilles Heel of fruit flies. To address this possibility, we generated transgenic flies that express yeast fRMsr^24,25^. These flies efficiently reduced free Met-*R*-SO, and we further employed this model to examine the role of this process in lifespan control and other processes.

## Materials and Methods

### DNA constructs, transgenic fly lines, and genetic backcross

The gene coding for fRMsr (YKL069W, Gene ID: 853794) was amplified from yeast genomic DNA with 5′-GCGGCCGCTCTCAAAACTATTATTTAAAGACACATGATTTATT-3′ and 5′-GCCTCGAGATGGGCTCATCAACCGGGTT-3′ primers and then cloned into the NotI/XhoI restriction sites of pUAST vector. Germline cell co-injection with the recombinant pUAST vector containing yeast fRMsr gene and the P{2-3} plasmid carrying transposase and balancing were used to obtain transgenic flies. By using this method, 6 *Drosophila* lines (1.1; yeast fRMsr gene on 3^rd^ chromosome, 2.1; 3^rd^ chromosome, 3.1; 2^nd^ chromosome, 4.2; 3^rd^ chromosome, 5.1; 2^nd^ chromosome, 9.1; 2^nd^ chromosome) were obtained. Among the 6 *Drosophila* lines carrying *UAS-fRMsr* in either 2^nd^ or 3^rd^ chromosome, 3 *Drosophila* lines (2.1 described as *fRMsr2.1*, 9.1 described as *fRMsr9.1*, 4.2 described as *fRMsr4*.2) produced viable progeny, whose fRMsr expression was induced by GAL4 expression, and thus were chosen for further experiments. The *GAL4* activator lines used in this study, *da-GAL4* [w*;; P{w^+*mW.hs*^ = *GAL4-da.G32)UH1}*] (stock #5460) and balancers were obtained from the Bloomington *Drosophila* Stock Center. A *fatbody-GAL4* driver [y*w*; P(w^+*mW.hs*^ = *GawB)FB P(w*^+m*^UAS-GFP 1010T2)] was obtained from R.P. Kühnlein (Max-Planck-Institut für Biophysikalische Chemie, Göttingen, Germany)^23^. Yellow-body white-eyes flies *yw* used as a wild type control were kindly provided by Dr. R. S. Sohal (University of Southern California). Then, three homozygous transgenic lines (*fRMsr2.1*, *fRMsr9.1*, and *fRMsr4.2*) and *GAL4* driver lines were backcrossed at least six times to *yw* to make the equivalent genetic backgrounds. After complete backcross of individual strain to *yw*, they were used for crosses to generate the following progeny: *da-GAL4* > *fRMsr2.1*, *da-GAL4* > *fRMsr9.1*, *da-GAL4* > *fRMsr4.2*, *da-GAL4* > *yw*, *yw* > *fRMsr2.1*, *yw* > *fRMsr9.1*, *yw* > *fRMsr4.2*.

### Diets and husbandary

For maintanence of flies and lifespan asays, three types of diet were used. First, it was the regular corn meal diet, composed of 85.7 g corn meal (Quaker Oats Company), 50 ml golden A unsulfured molasses (Groeb Farms Inc), 71.4 g Torula yeast (MP Biomedicals), 2.86 g p-hydroxybenzoic acid methyl ester (Sigma), 6.4 g agar (MoorAgar Inc) and 5.7 ml propionic acid (Sigma) per liter of water^23,26^. This diet was used for maintaining fly strains, counting pupa numbers, and carrying out lifespan analyses. Second, we used a regular diet supplied by the Harvard Medical School fly kitchen. Briefly, 12.65 g yeast, 7.33 g soy flour, 53.45 g cornmeal, 4.23 g agar, 4.23 g maltose, 56 ml corn syrup, 3 ml propionic acid, and 10 ml tegosept per liter of water were used^27^. This diet was used to examine lifespan of given fly strains. Third, we used a chemically defined diet^28,29^. Briefly, 101.07 g Diet TD.10417 (Harlan Teklad), 100 mg lecitin from soybean (Sigma), 500 mg ribonucleic acid from Torula yeast (Sigma), 100 g of dextrose, 20 g agar, 2.85 ml propionic acid, 0.255 ml of phosphoric acid (Sigma) and indicated amounts of Met (Sigma) per liter of water were used. This diet was used to examine the effect of Met levels on lifespan. All fly lines were grown on the corn meal diet mentioned above. Vials were kept with less than 50–60 eggs and then the hatched flies were transferred to one of the two types of fresh corn meal food. After mating for 1–2 days, three day old mated flies were collected using etherization, sorted by sex and used for subsequent experiments. To maintain fly strains, they were raised on corn meal food and transferred to fresh vials without anesthesia every three days. Flies were kept in a temperature-controlled chamber at 25 °C with 12 h light/dark cycle and approximately 60% humidity for all experiments.

### Lifespan analysis

Newly hatched flies were sorted for sex (within 16 h) at 18  °C and then held on the regular corn meal diet with less than 40 flies per vial. The same number of male and female flies were incubated for 30 h for mating. Then, the flies were sorted for sex again and kept on the regular corn meal diet until the use. For lifespan studies, less than 50 male or female 3-day-old flies were placed in plastic cup cages, a system which we and others have routinely used for aging assays^30^. Fresh food (regular or defined diet) was supplied and dead flies were removed from each cage every 3 days until all flies died. Control and experimental group trials were always performed concurrently under the same conditions. Flies were maintained in a temperature-controlled incubator at 25 °C with 12-h light/dark cycle and 50–60% humidity.

### Paraquat resistance test

Paraquat resistance tests were performed as described previously with minor modifications^31,32^. Briefly, fly media containing 1.3% low melting agarose, 1% sucrose, and 10 mM paraquat with or without 1 mM Met were prepared for paraquat resistance tests. 10 mM paraquat was added at 45 °C to avoid loss of activity. Male flies were prepared as described above, and then 15-day-old flies were sorted in groups of 15 flies per vial 3 days prior to the test. On the day of the test, after starvation for 6 h, flies were transferred without etherization to vials with the paraquat-containing media. The numbers of dead flies were counted every 12 h until all flies were dead. 4 replicates were performed for each control and experimental group in a temperature-controlled incubator at 25 °C with 12-h light/dark cycle and 50–60% humidity. Survival was analyzed by Log-rank test using the JMP (version 10) software (SAS Institute Inc.).

### Starvation resistance test

Male flies were prepared as described above, and then 3-day-old flies were sorted in groups of 15 flies per vial. 4 replicates were prepared for each control and experimental group. Male flies were maintained on the regular corn meal diet for 18 days prior to the test. All flies were starved in vials with 6 ml 1% agar and kept in 12 h light/dark cycle at 25 °C. Total number of flies tested for each genotype was 60. Dead animals were counted every 12 h.

### Pupa production

Newly hatched flies were sorted for sex within 16 h at 18 °C and then 5 females were incubated with 5 males to count on pupa production during 24 h. We had 5–10 replicas of each control and experimental group. Females laid eggs for 24 h, then flies were transferred to new vials and the number of pupa was counted after enclosion.

### Food consumption

Newly hatched female flies were prepared and reared on the regular corn meal diet as described above. For the feeding assay, 0.1% (w/v) erioglaucine (FD&C Blue no. 1, Sigma) was added to each diet that contains 1.3% low melting agarose and 1% sucrose. 6- and 24-day-old flies were fed this diet for 30 min after short-term starvation (12 noon–12:30 pm) as described^29,33^. Then, flies were immediately collected in Eppendorf tubes by snap freezing in liquid nitrogen. Five female flies per group (10–20 groups for each control and experiemental strain were used for a 30-min incubation) were homogenized in 200 μl of phosphate-buffered saline, and the homogenate was centrifuged at 13,000 r.p.m. for 15 min. Absorbance of the supernatant was measured at 625 nm, with 675 nm used as a reference, and then compared with the absorbance among the tested strains to calculate consumed food mass.

### Western blotting and methionine sulfoxide reduction activity assays

Six-day-old flies were collected and snap frozen in liquid nitrogen. 30 flies were homogenized with disposable plastic homogenizer in Effendorf tube in PBS buffer (pH = 7.4). After centrifugation at 4 °C at 13,000 rpm for 15 min, supernant was collected for further Western blotting and methionine sulfoxide reduction activity assays. For Western blotting, 120 µg of total protein lysate was electrophoresed on NuPAGE® Novex 10% Bis-Tris gels (Invitrogen), transferred onto PVDF membranes and immunoblotted with antibodies specific to yeast fRMsr^15^. Finally, fRMsr expression was visualized by using the ECL detection system (Sigma).

For protein-based Met sulfoxide reduction activity assays, dabsyl-Met-*R*-SO and dabsyl-Met-*S*-SO were prepared as previously described^15^, and then the reaction mixture (100 µl) containing 20 mM dithiothreitol, 200 µM dabsyl-Met-*R*-SO (or 200 µM dabsyl-Met-*S*-SO), and tissue lysate containing 200 ~ 400 µg of total protein in PBS (pH 7.4) was prepared. The reaction was carried out at 37 °C for 30 min and stopped by adding 200 µl of acetonitrile at 4 °C for 10 min. After centrifugation at 13,000 rpm at 4 °C for 30 min, supernatant was collected, that contained dabsyl-Met. Then, 50 µl of supernatant were injected onto a C_18_ column (ZORBAX Eclipse XDB-C18, Agilent Technologies) to quantify dabsyl-Met at 436 nm using an HPLC instrument with a UV detector^34^.

For free Met sulfoxide reduction activity assay, free Met, Met-*R*-SO and Met-*S*-SO were prepared as previously described^15^, and the reaction mixture (100 µl) contained 20 mM dithiothreitol, 200 µM free Met-*R*-SO (or 200 µM free Met-*S*-SO), and 200–400 µg of total protein in PBS (pH 7.4). Reactions were carried out at 37 °C for 30 min and stopped by adding 200 µl acetonitrile at 4 °C for 10 min. After centrifugation at 13,000 rpm at 4 °C for 30 min, supernatant was collected that contained free Met. The supernatant (50 μl) was diluted 10 times with distilled water and prepared for OPA derivatization. OPA derivatization of amino acids and HPLC analysis were performed as described^35^ with minor modifications. The derivatization reagent was freshly prepared as a stock solution (40 mg of *o*-phthalaldehyde, 1 ml of methanol, 50 μl of 2-mercaptoehanol, 5 ml of 0.1 M Na_2_B_4_O_7_, pH 9.5) at room temperature in a capped amber vial. Sample solutions (2–5 μl) were mixed with the OPA derivatization reagent to a 100 μl final volume. Following a 2 min reaction at room temperature, the mixture was injected onto a C_18_ column (ZORBAX Eclipse XDB-C18, Agilent Technologies) to quantify free Met. Detection was by fluorescence of Met derivatives using a Waters 474 scanning fluorescence detector with excitation at 330 nm and emission at 445 nm.

### Measurement of oxidized and reduced glutathione levels

We measured oxidized (GSSG) and reduced (GSH) glutathione levels as previously described^36,37^. 12- and 51-day-old flies were prepared and immobilized on ice for 1–2 min. After weighing, they were homogenized in 10 volumes of freshly prepared iced-cold 5% (w/v) MPA using a 1.5 ml micricentrifuge tube, a Teflon micropestle, and a handheld motor. The homogenates were incubated for 30 min on ice and centrifuged at 13,000 r.p.m. for 30 min at 4 °C. Then, the supernatants were filtered using 0.45 µm PTFE Acordisc CR 4 mm syringe filters. The filtrates (50 µl for GSH and 200 µl for GSSG) were transferred to vials and stored at – 80 °C for up to 1 month. For GSH analysis, 0, 0.5, 1, and 5 µM GSH standards or 50 µl of the supernatant were added to 1 ml of 0.1% EDTA in 0.1 M sodium hydrogenphosphate (pH 8.0). Then, 20 µl mixture was added to 300 µl of 0.1% EDTA in 0.1 M sodium hydrogenphosphate and 20 µl of 0.1% OPA in methanol. Capped tubes were incubated at 25 °C for 3 min in dark, and then the contents were filtered through 0.2 µm nylon filters and injected onto a C_18_ column (ZORBAX Eclipse XDB-C18, Agilent Technologies) for further HPLC analysis. For GSSG analysis, 0, 0.5, 1, and 5 µM GSSG standards or 200 µl supernatant were incubated at 25 °C with 200 µl of 40 mM NEM for 25 min in dark and then this mixture was added to 750 µl of 0.1 M NaOH. After then, 20 µl of this mixture was added to 300 µl of 0.1 M NaOH and 20 µl of 0.1% OPA in methanol. Capped tubes were incubated at 25 °C for 3 min in dark, and then the contents were filtered through 0.2 µm nylon filters and injected onto a C_18_ column (ZORBAX Eclipse XDB-C18, Agilent Technologies) for further HPLC analysis. Isocratic analysis was performed with 15% methanol in 25 mM sodium hydrogenphosphate (v/v), pH 6.0, as a mobile phase. Detection was by fluorescence using a Waters 474 scanning fluorescence detector with excitation at 350 nm and emission at 420 nm.

### Statistical analyses

Statistical analyses were performed using the JMP (version 10) software (SAS Institute Inc.). Enzyme activities (overexpression versus control), food consumption at two different ages, and pupa production at each time point were analyzed by Student’s *t* test. *Drosophila* survival analysis on various diets and comparison of survivorship curves were carried out using the Log-rank and Wilcoxon tests.

## Results

### Ectopic expression of yeast fRMsr in *Drosophila* supports the reduction of free methionine-*R*-sulfoxide

**Figure 1:**
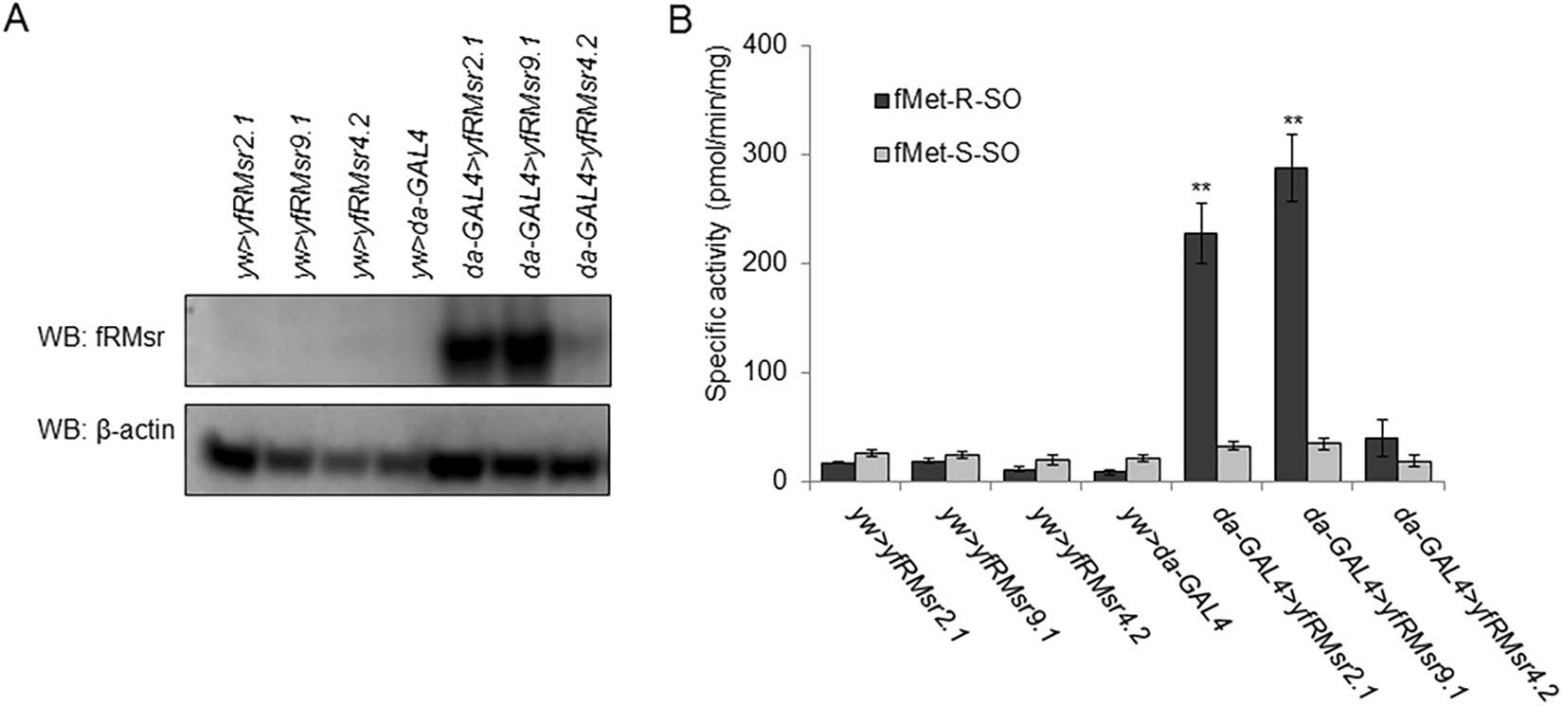
Characterization of transgenic flies expressing yeast fRMsr. (**A**) Western blotting to examine the expression of fRMsr and (**B**) specific activity for the reduction of free Met-*R*-SO in *da-GAL4* > *fRMsr2.1*, *da-GAL4* > *fRMsr9.1*, *da-GAL4* > *fRMsr4.2*, *da-GAL4* > *yw*, *yw* > *fRMsr2.1*, *yw* > *fRMsr9.1*, *yw* > *fRMsr4.2* lines. fMet-R-SO, fMet-S-SO, pMet-R-SO, pMet-S-SO represent free Met-R-SO, free Met-S-SO, protein-based Met-R-SO, and protein-based Met-S-SO, respectively (*P < 0.05, **P < 0.01, Student’s *t*-test).

Mouse MsrA is known to efficiently reduce free Met-*S*-SO, whereas the activity of mouse MsrBs towards the reduction of free Met-*R*-SO is low, leading to the accumulation of free Met-*R*-SO in mouse plasma^15^. *Drosophila* contains single MsrA and MsrB genes and thus is also expected to exhibit low activity with free Met-*R*-SO. To restore the ability of flies to reduce free Met-*R*-SO reduction, which was lost during evolution of metazoa, we generated transgenic flies expressing yeast fRMsr by using the UAS-GAL4 binary system, which is commonly used to study gene expression and function in *Drosophila*^38^. Three independent homozygous UAS responder lines, *fRMsr2.1*, *fRMsr9.1*, and *fRMsr4.2*, were prepared that express yeast fRMsr by using this system. These responder lines and the activator lines (*da-GAL4* for whole body expression and *fatb-GAL4* for fat-body specific expression) were backcrossed with the control *yw* line at least 6 times. Then, the progeny of the cross among the three responder lines, the activator lines, and the control *yw* line were subjected to Western blotting (Fig. 1A and Supplementary Figure 1A) and free Met-*R*-SO reduction activity assays (Fig. 1B and Supplementary Figure 1B). fRMsr was highly expressed in *da-GAL4* > *fRMsr2.1* and *da-GAL4* > *fRMsr9.1* lines, expressed at a low level in *da-GAL4* > *fRMsr4.2* line, and was not detected in any of the control lines. With respect to crossing with the *fatb-GAL4* activator line, although fRMsr expression was reduced in *fatb-GAL4* > *fRMsr2.1* and *fatb-GAL4* > *fRMsr2.1* lines, its expression pattern was consistent with the results of the cross with *da-GAL4* activator line (Supplementary Figure 1A). The fRMsr catalytic activity agreed with the Western blotting-based expression analysis, i.e. catalytic activities of the *da-GAL4* > *fRMsr2.1* and *da-GAL4* > *fRMsr9.1* lines were ~222 pmol/min/mg and ~284 pmol/min/mg, respectively, whereas the activity in the *da-GAL4* > *fRMsr4.2* line was 6–7 fold lower. Also, catalytic activities of the *fatb-GAL4* > *fRMsr2.1* and *fatb-GAL4* > *fRMsr2.1* lines were ~55 pmol/min/mg and ~57 pmol/min/mg, respectively, whereas the activity of the *da-GAL4* > *fRMsr4.2* line was ~23 pmol/min/mg (Fig. 1B and Supplementary Figure 1B).

### Expression of fRMsr extends lifespan

**Figure 2:**
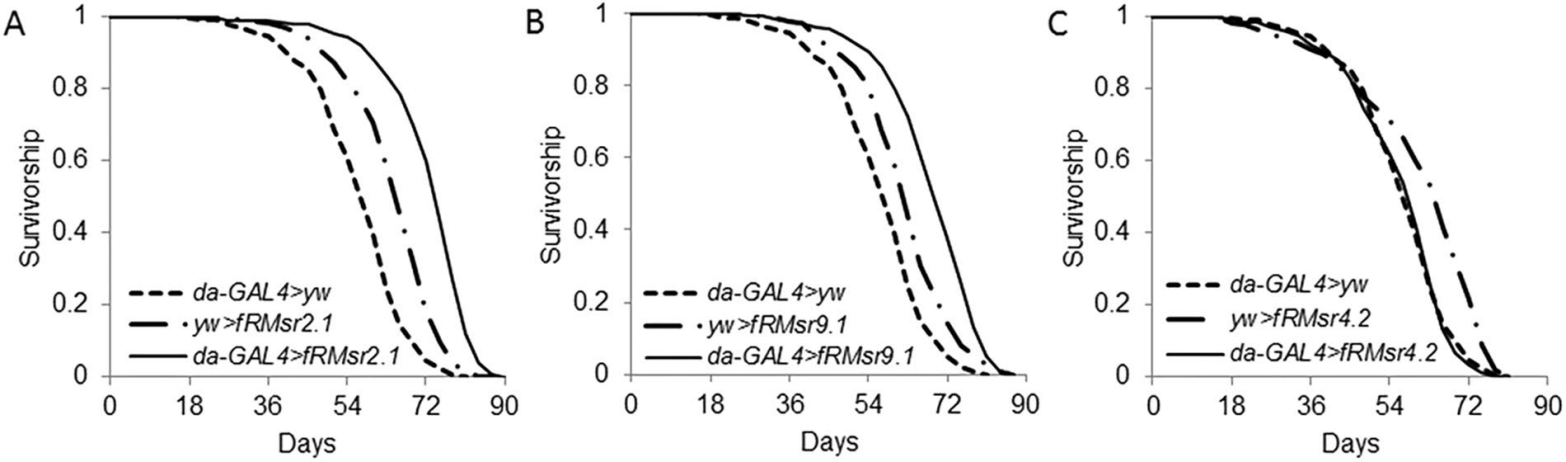
Survivorship of transgenic male flies expressing yeast fRMsr on a regular corn meal diet. (**A**) *da-GAL4* > *fRMsr2.1* and its two controls, *da-GAL4* > *yw* and *yw* > *fRMsr2.1*, or (**B**) *da-GAL4* > *fRMsr9.1* and its two controls, *da-GAL4* > *yw* and *yw* > *fRMsr9.1*, or (**C**) *da-GAL4* > *fRMsr4.2* and its two controls, *da-GAL4* > *yw* and *yw* > *fRMsr4.2*, were analayzed. All lifespan assays were performed nine times independently. See Methods for composition of the regular corn meal diet and Table 1 for summary statistics.

MsrA can support resistance to oxidative stress by reducing both protein-based and free Met sulfoxide, and it can also regulate protein function via protein-based Met oxidation/reduction^39^, but it is not fully understood which of these functions underlies its contribution to lifespan extension upon overexpression in fruit flies. The fRMsr lines, *da-GAL4* > *fRMsr2.1*, *da-GAL4* > *fRMsr9.1*, and *da-GAL4* > *fRMsr4.2*, which only support free Met sulfoxide reduction, were examined for lifespan (Fig. 2A–C and Table 1). Interestingly, mean lifespan of *da-GAL4* > *fRMsr2.1* and *da-GAL4* > *fRMsr9.1* lines was increased compared to control lines (*da-GAL4* > *fRMsr2.1* vs *da-GAL4* > *yw*, 28%, *p* < 0.0001; *da-GAL4* > *fRMsr2.1* vs *yw* > *fRMsr2.1*, 13%, *p* < 0.0001; *da-GAL4* > *fRMsr9.1* vs *da-GAL4* > *yw*, 20%, *p* < 0.0001; *da-GAL4* > *fRMsr9.1* vs *yw* > *fRMsr9.1*, 10%, *p* < 0.0001, Log-Rank test). On the other hand, the *da-GAL4* > *fRMsr4.2* line, which expressed fRMsr at a low level, showed no lifespan extension (Fig. 2C and Table 1). Finally, we performed the lifespan assay using virgin female and male flies expressing fRMsr to test whether the fRMsr-dependent lifespan extension was dependent on Met or influenced by reproduction. As in the case of the mated male flies mentioned above, mean lifespan of *da-GAL4* > *fRMsr2.1* and *da-GAL4* > *fRMsr9.1* lines was increased compared to control lines for both virgin female and male flies (virgin female: *da-GAL4* > *fRMsr2.1* vs *da-GAL4* > *yw*, 18%, *p* < 0.0001; *da-GAL4* > *fRMsr2.1* vs *yw* > *fRMsr2.1*, 16%, *p* < 0.0001; *da-GAL4* > *fRMsr9.1* vs *da-GAL4* > *yw*, 18%, *p* < 0.0001; *da-GAL4* > *fRMsr9.1* vs *yw* > *fRMsr9.1*, 13%, *p* < 0.0001, male: *da-GAL4* > *fRMsr2.1* vs *da-GAL4* > *yw*, 27%, *p* < 0.0001; *da-GAL4* > *fRMsr2.1* vs *yw* > *fRMsr2.1*, 17%, *p* < 0.0001; *da-GAL4* > *fRMsr9.1* vs *da-GAL4* > *yw*, 19%, *p* < 0.0001; *da-GAL4* > *fRMsr9.1* vs *yw* > *fRMsr9.1*, 11%, *p* = 0.0005, Log-Rank test) (Supplementary Table 1). Therefore, the data suggests an association of elevated free Met-*R*-SO reduction with lifespan extension, and this association is supported by experiments with both virgin female and male flies.

**Table 1:** Lifespan of male flies expressing fRMsr on a regular corn meal diet and test of homogeneity of survivorship.

GB	Sex	Lifespan			Log-Rank		Wilcoxon		% increase of mean lifespan from control
		Number of flies	Mean ± s.e.m.	Median	χ^2^	ρ > χ^2^	χ^2^	ρ > χ^2^	
*da-GAL4* > *yw*	M	370	57.10 ± 0.58	58.5	347.85	<0001^*^	301.76	<0.0001^*^	28
*da-GAL4* > *fRMsr2.1*	M	309	73.07 ± 0.59	75					
*yw* > *fRMsr2.1*	M	319	64.83 ± 0.59	66	131.24	<0001^*^	119.98	<0.0001^*^	13
*da-GAL4* > *fRMsr2.1*	M	309	73.07 ± 0.59	75					
*da-GAL4* > *yw*	M	370	57.10 ± 0.58	58.5	202.57	<0001^*^	181.99	<0.0001^*^	20
*da-GAL4* > *fRMsr9.1*	M	328	68.63 ± 0.60	69					
*yw* > *fRMsr9.1*	M	297	62.51 ± 0.62	63	55.51	<0001^*^	62.57	<0.0001^*^	10
*da-GAL4* > *fRMsr9.1*	M	328	68.63 ± 0.60	69					
*da-GAL4* > *yw*	M	370	57.10 ± 0.58	58.5	0.075	0.7848	0.0001	0.9937	−1
*da-GAL4* > *fRMsr4.2*	M	301	56.69 ± 0.67	60					
*yw* > *fRMsr4.2*	M	292	61.01 ± 0.87	66	54.33	<0.0001	32.12	<0.0001	−7
*da-GAL4* > *fRMsr4.2*	M	301	56.69 ± 0.67	60					

GB: Genetic background.

^*^Statistically significant difference.

^**^% increase of mean lifespan over flies on the control diet.

**Figure 3:**
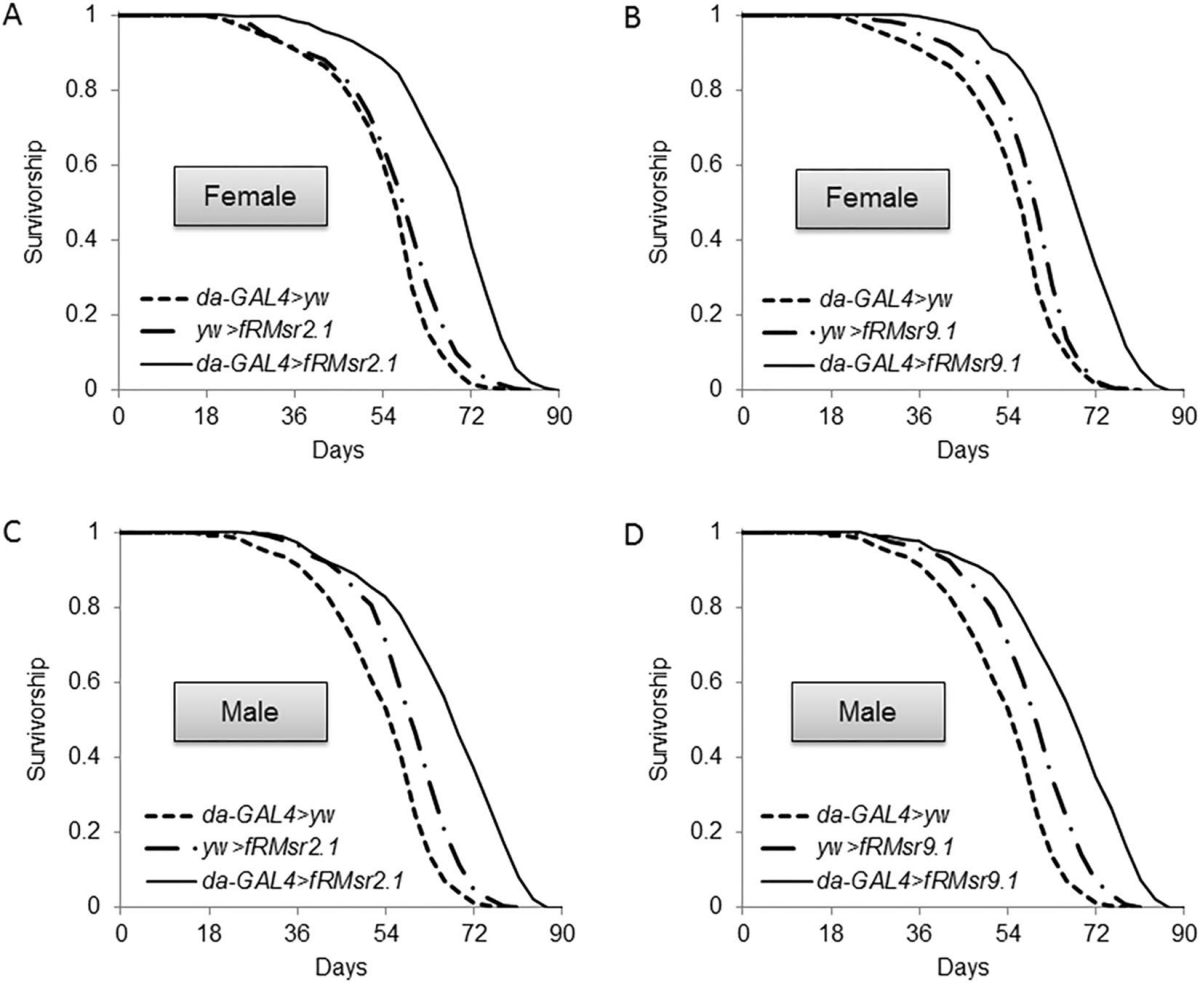
Survivorship of mated female and male flies expressing yeast fRMsr on another type of regular diet. Female flies of (**A**) *da-GAL4* > *fRMsr2.1* and its two controls, *da-GAL4* > *yw* and *yw* > *fRMsr2.1*, or (**B**) *da-GAL4* > *fRMsr9.1* and its two controls, *da-GAL4* > *yw* and *yw* > *fRMsr9.1*, were analyzed. Male flies of (**C**) *da-GAL4* > *fRMsr2.1* and its two controls, *da-GAL4* > *yw* and *yw* > *fRMsr2.1*, or (**D**) *da-GAL4* > *fRMsr9.1* and its two controls, *da-GAL4* > *yw* and *yw* > *fRMsr9.1*, were analyzed. All lifespan assays were performed nine times independently. See Methods for composition of the diet and Supplementary Table 2 for summary statistics.

To examine gender and diet effects on lifespan extension by fRMsr, we subjected transgenic flies to lifespan analysis using different diets. The outcome of lifespan assays may be affected by diet. As such, we used another regular diet that was supplied by the Harvard Medical School fly kitchen and also has been used for lifespan assay (see Materials and Methods)^40^. The lifespan of mated females and males flies of *da-GAL4* > *fRMsr2.1* and *da-GAL4* > *fRMsr9.1* genotypes was extended. The lifespan extension of females ranged from 15 to 24% (*da-GAL4* > *fRMsr2.1* vs *da-GAL4* > *yw*, 24%, *p* < 0.0001; *da-GAL4* > *fRMsr2.1* vs *yw* > *fRMsr2.1*, 19%, *p* < 0.0001; *da-GAL4* > *fRMsr9.1* vs *da-GAL4* > *yw*, 24%, *p* < 0.0001; *da-GAL4* > *fRMsr9.1* vs *yw* > *fRMsr9.1*, 16%, *p* < 0.0001, Log-Rank test) and the lifespan extension of males ranged from 12 to 25% (*da-GAL4* > *fRMsr2.1* vs *da-GAL4* > *yw*, 25%, *p* < 0.0001; *da-GAL4* > *fRMsr2.1* vs *yw* > *fRMsr2.1*, 13%, *p* < 0.0001; *da-GAL4* > *fRMsr9.1* vs *da-GAL4* > *yw*, 25%, *p* < 0.0001; *da-GAL4* > *fRMsr9.1* vs *yw* > *fRMsr9.1*, 12%, *p* < 0.0001, Log-Rank test) (Fig. 3 and Supplementary Table 2). Thus, fRMsr expression  extends lifespan regardless of gender and diet type.

### Expression of fRMsr does not affect starvation resistance, fecundity, and food consumption

**Figure 4:**
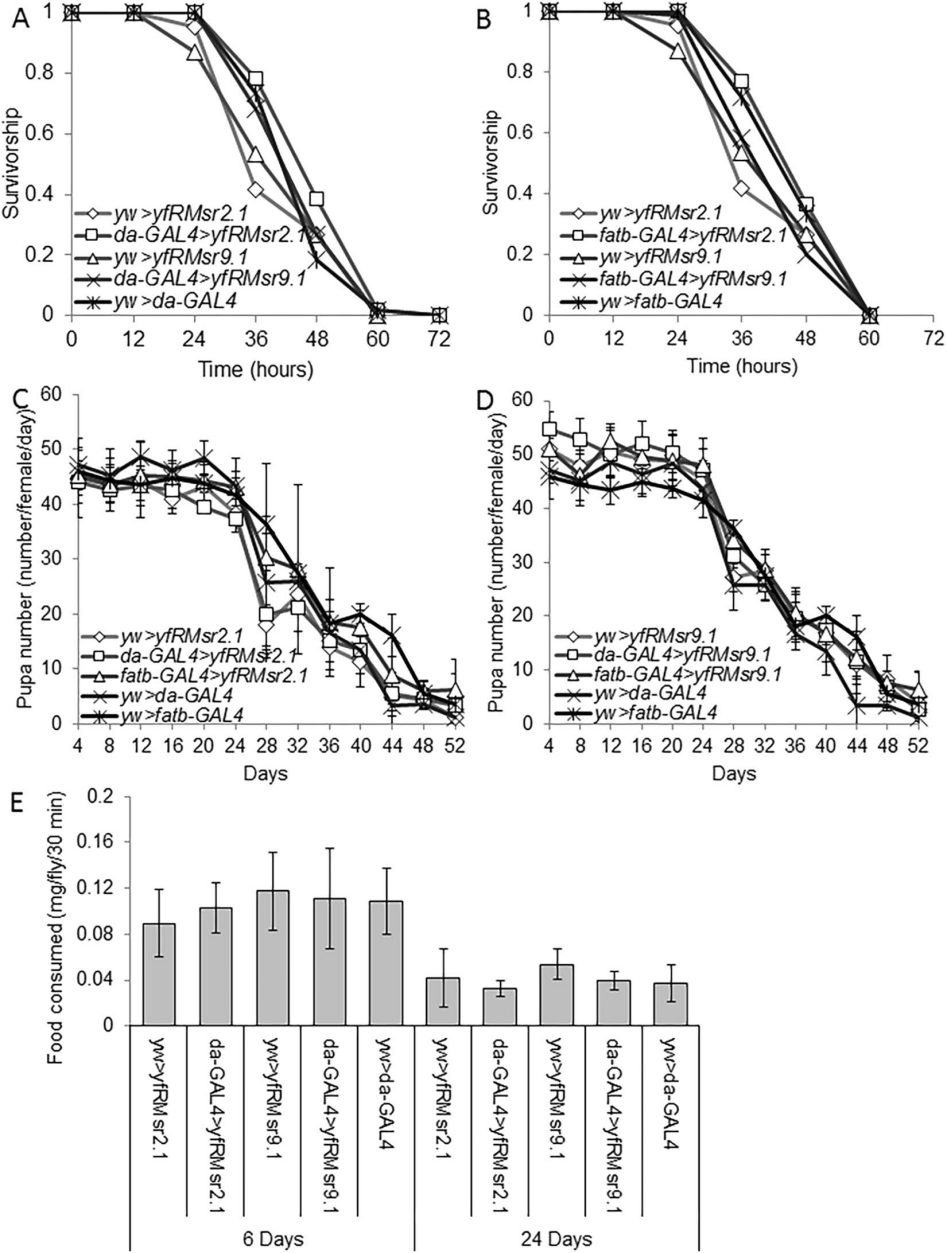
Starvation resistance, pupa production, and food consumption of fRMsr expressing flies. Survivorship of (**A**) *da-GAL4* > *fRMsr2.1*, *da-GAL4* > *fRMsr9.1*, *da-GAL4* > *yw*, *yw* > *fRMsr2.1*, and *yw* > *fRMsr9.1* strains or (**B**) *fatb-GAL4* > *fRMsr2.1*, *fatb-GAL4* > *fRMsr9.1*, *yw* > *fatb-GAL4*, *yw* > *fRMsr2.1*, and *yw* > *fRMsr9.1* strains was examined every 12 h, and the flies were analyzed for resistance to starvation (Log-rank test). Pupa production, expressed as a number per female per day of (**C**) *da-GAL4* > *fRMsr2.1*, *da-GAL4* > *fRMsr9.1*, *da-GAL4* > *yw*, *yw* > *fRMsr2.1*, and *yw* > *fRMsr9.1* strains or (**D**) *fatb-GAL4* > *fRMsr2.1*, *fatb-GAL4* > *fRMsr9.1*, *yw* > *fatb-GAL4*, *yw* > *fRMsr2.1*, and *yw* > *fRMsr9.1* strains was analyzed every 4 days until flies were 52-day-old, and the reproduction rate at each time point was examined (Student’s *t*-test). (**E**) Flies were reared on the regular corn meal diet and then collected on the 6th or 24th days. Food consumption of *da-GAL4* > *fRMsr2.1*, *da-GAL4* > *fRMsr9.1*, *da-GAL4* > *yw*, *yw* > *fRMsr2.1*, and *yw* > *fRMsr9.1* strains for 30 min was measured on the 6th day (mean ± s.d., *n* = 100~200) and 24th day (mean ± s.d., *n* = 100~200) via the feeding assay using erioglaucine. Then, food consumption rate was calculated at two different time points (Student’s *t*-test).

Increased lipid content of adult flies is known to enhance resistance to starvation and is often associated with lifespan extension^40^. By reducing free Met-*R*-SO, fRMsr might supply additional Met which may participate in cell metabolism. Consequently, it is possible that excessive energy may promote body fat synthesis as an energy storage form. We examined starvation resistance of transgenic flies expressing fRMsr in whole body or fat body. *fRMsr2.1* or *fRMsr9.1* lines were crossed with the *da-GAL4* or *fatb-GAL4* drivers and the progeny were subjected to the analysis of starvation resistance (Fig. 4A,B). No significant difference was observed among the tested strains.

Cost of reproduction may hinder investment to survivorship, adversely affecting life expectancy. To examine how reproduction is affected by fRMsr expression, we analyzed pupa production in the strains used in the starvation test. The number of pupa produced following one day incubation of five males and five females was counted for two months after birth (Fig. 4C,D). No difference in pupa production rate was observed among the tested lines.

Calorie restriction may affect lifespan, but the effect depends on the amount of consumer nutrients and other factors. To examine a potential effect of changes in food consumption upon fRMsr expression, 6-day old and 24-day old flies of *da-GAL4* > *yfRMsr2.1*, *da-GAL4* > *yfRMsr9.1*, *yw* > *fRMsr2.1*, *yw* > *fRMsr9.1*, and *da-GAL4* > *yw* lines were examined for food consumption that was measured following 30 min incubation on the diet containing erioglaucine (Fig. 4E). Again, no difference was observed among the strains.

### Methionine supports oxidative stress resistance of fRMsr flies

**Figure 5:**
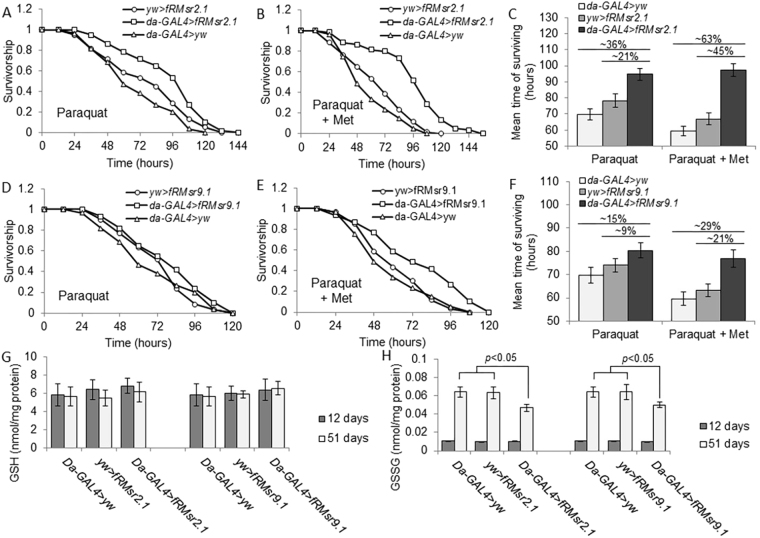
Paraquat resistance of transgenic flies expressing fRMsr and its dependence on Met availability. Survivorship curve of *da-GAL4* > *fRMsr2.1* and its two controls, *da-GAL4* > *yw* and *yw* > *fRMsr2.1*, reared on the diet containing agar, sugar, and 10 mM paraquat (**A**) without or (**B**) with 1 mM Met is shown, and (**C**) mean time of survivorship was analyzed (mean ± s.d., *n* = 60, Log-rank test). Survivorship curve of *da-GAL4* > *fRMsr9.1* and its two controls, *da-GAL4* > *yw* and *yw* > *fRMsr9.1*, reared on the diet containing agar, sugar, and 10 mM paraquat (**D**) without or (**E**) with 1 mM Met is shown, and (**F**) mean time of survivorship was analyzed (mean ± s.d., *n* = 60, Log-rank test). All paraquat resistance tests were performed 4 times independently. See Supplementary Table 3 for summary statistics. 12- and 51-day-old flies of the *da-GAL4* > *fRMsr2.1*, *da-GAL4* > *fRMsr9.1*, *yw* > *fRMsr2.1*, *yw* > *fRMsr9.1*, and *da-GAL4* > *yw* lines were subjected to the analyses of (**G**) reduced glutathione (GSH) and (**H**) oxidized glutathione (GSSG) levels. These experiments were performed three times independently (mean ± s.d., student’s *t*-test).

fRMsr may support removal of ROS associated with free methionine, but does this function contribute to lifespan extension upon fRMsr expression? To address this question, 18-day-old flies of *da-GAL4* > *fRMsr2.1*, *da-GAL4* > *fRMsr9.1*, *da-GAL4* > *fRMsr4.2*, *yw* > *fRMsr2.1*, *yw* > *fRMsr9.1*, *yw* > *fRMsr4.2*, and *da-GAL4* > *yw* lines were reared on the sugar-agar diet containing 10 mM paraquat to increase ROS production (Fig. 5 and Supplementary Table 3). Under these conditions, *da-GAL4* > *fRMsr2.1* and *da-GAL4* > *fRMsr9.1* showed increased survivorship, compared with control lines (No Met, *da-GAL4* > *fRMsr2.1* vs *da-GAL4* > *yw*, 36%, *p* < 0.0001; *da-GAL4* > *fRMsr2.1* vs *yw* > *fRMsr2.1*, 21%, *p* = 0.0064; *da-GAL4* > *fRMsr9.1* vs *yw* > *da-GAL4*, 15%, *p* = 0.064; *da-GAL4* > *fRMsr9.1* vs *yw* > *fRMsr9.1*, 9%, *p* = 0.057, Log-Rank test). Moreover, when 1 mM Met was added to the same diet containing 10 mM paraquat, survivorship of *da-GAL4* > *fRMsr2.1* and *da-GAL4* > *fRMsr9.1* was further extended (1 mM Met, *da-GAL4* > *fRMsr2.1* vs *da-GAL4* > *yw*, 63%, *p* < 0.0001; *da-GAL4* > *fRMsr2.1* vs *yw* > *fRMsr2.1*, 45%, *p* < 0.0001; *da-GAL4* > *fRMsr9.1* vs *da-GAL4* > *yw*, 29%, *p* < 0.0001; *da-GAL4* > *fRMsr9.1* vs *yw* > *fRMsr9.1*, 21%, *p* = 0.0003, Log-Rank test) (Fig. 5A–F and Supplemetary Table 4). The sugar-yeast diet contains no free Met, and we did not observe an increased survivorship of *da-GAL4* > *fRMsr2.1* and *da-GAL4* > *fRMsr9*.1 lines on this diet without paraquat (data not shown). Thus, fRMsr expression in transgenic flies supports increased survivorship against paraquat-induced oxidative stress on this sugar-yeast diet. Next, we measured GSH and GSSG levels in 12- and 51-day-old flies in order to estimate oxidative stress levels in young and old flies upon fRMsr expression. In 12-day-old flies, we did not observe a difference in GSH and GSSG, whereas GSSG levels were reduced in 51-day-old flies of *da-GAL4* > *fRMsr2.1* and *da-GAL4* > *fRMsr9.1* strains, when compared with other control strains, but GSH levels were not changed (Fig. 5G,H). Accordingly, this finding shows that fRMsr expression alleviates oxidative stress and thus reduced accumation of GSSG. Taken together, ectopic expression of yeast fRMsr conferred enhanced oxidative stress resistance, and this effect was amplified by dietary Met.

### Lifespan extension by fRMsr depends on Met concentration

**Figure 6:**
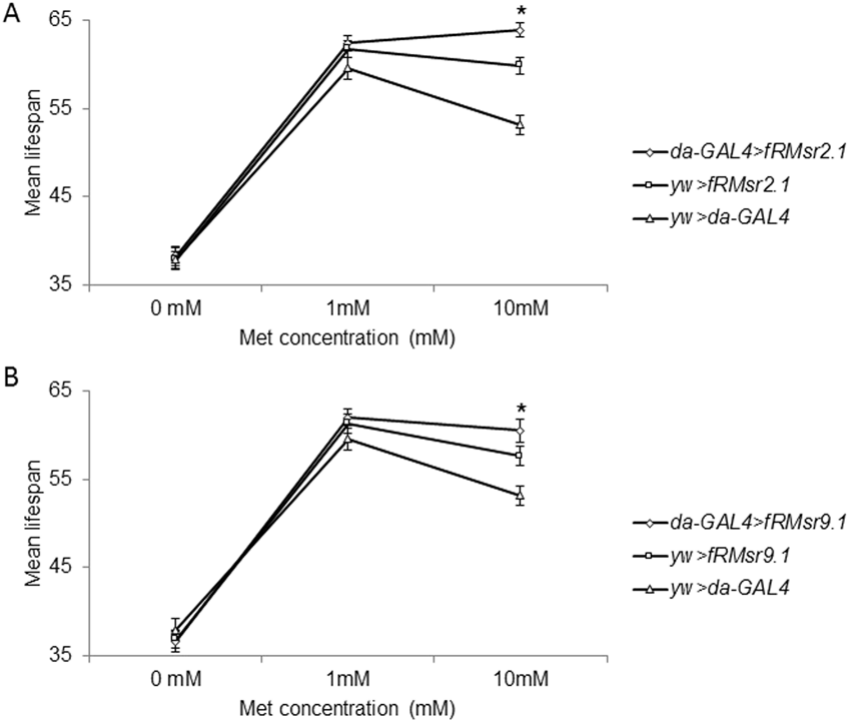
Mean lifespan of transgenic male flies expressing fRMsr on the chemically defined diet containing various Met concentrations. Mean lifespan of (**A**) *da-GAL4* > *fRMsr2.1* and its two controls, *da-GAL4* > *yw* and *yw* > *fRMsr2.1*, or (**B**) *da-GAL4* > *fRMsr9.1* and its two controls, *da-GAL4* > *yw* and *yw* > *fRMsr9.1*, reared on the chemically defined diet containing 0, 1, or 10 mM Met was determined. (mean ± s.e.m., *n* = 88~123, *P < 0.05, Log-rank test). All lifespan assays were performed three times independently. See Methods for composition of the chemically defined diet and Supplementary Table 4 for summary statistics .

We hypothesized that Met may be a factor that supports lifespan extension by fRMsr by removing ROS. To test this possibility, two fRMsr-expressing strains, *da-GAL4* > *fRMsr2.1*, *da-GAL4* > *fRMsr9.1*, and control strains, *yw* > *fRMsr2.1*, *yw* > *fRMsr9.1*, and *da-GAL4* > *yw*, were raised on the chemically defined diet containing 0, 1, 10 or 100 mM Met and their survivorhip was determined (Fig. 6, Supplementary Figure 2 and Supplementary Table 4). This chemically defined diet was devised to investigate effects of individual dietary components^28,29^. We applied this diet to examine lifespan of male flies expressing fRMsr upon various Met concentrations. Lifespan extension upon fRMsr expression was observed on the diet containing 10 mM Met, but not on the 0 mM and 1 mM Met diet (10 mM Met, *da-GAL4* > *fRMsr2.1* vs *da-GAL4* > *yw*, 20%, *p* < 0.0001; *da-GAL4* > *fRMsr2.1* vs *yw* > *fRMsr2.1*, 7%, *p* = 0.0005; *da-GAL4* > *fRMsr9.1* vs *da-GAL4* > *yw*, 14%, *p* < 0.0001; *da-GAL4* > *fRMsr9.1* vs *yw* > *fRMsr9.1*, 3%, *p* = 0.0189, Log-rank test). In particular, we did not observe any lifespan extension in both *da-GAL4* > *fRMsr2.1* and *da-GAL4* > *fRMsr9.1* lines on the diet without Met. Thus, cooperation between Met and fRMsr was required for lifespan extension.

## Discussion

fRMsr occurs across most prokaryotes and unicellular eukaryotes, where it catalyzes the reduction of free Met-*R*-SO. However, this protein is absent in metazoa, which lost it during evolution, and this deficiency is not fully compensated for by other enzymes, leading to specific elevation of free Met-*R*-SO in mouse plasma^15^. In this study, we asked a simple question: can restoration of free Met-*R*-SO reduction in an animal support its physiological functions and regulate lifespan? Using fruit flies as a model, we found that ectopically expressed fRMsr increases stress resistance and extends lifespan of animals without affecting reproduction and food consumption. While lifespan extention by fRMsr was modest, we observed this effect in different transgenic lines as well as with different diets and with both virgin and mated flies. Interestingly, this effect was dependent on the levels of dietary Met: flies maintained on higher Met were better protected by fRMsr. Apparently, it is the reduction of free Met-*R*-SO that mediates the beneficial effects of the exogenous oxidoreductase lost during evolution and restored via ectopic expression in fruit flies.

fRMsr has an important function in metabolism (by providing free methionine from methionine sulfoxide) and oxidative stress resistance (by removing ROS through cyclic oxidation and reduction of methionine). Deletion of this gene reduces lifespan of yeast^14^, supporting its importance in lifespan control. What is then a possible evolutionary significance of its loss in metazoa? We hypothesize that unicellular organisms, including prokaryotes and lower eukaryotes, exhibit rapid growth if provided with necessary resources. However, feast may quickly turn to starvation due to rapid consumption. As such, unicellular organisms may face Met deficiency, in turn affecting the levels of S-adenosylmethionine, glutathione and other compounds dependent on Met supply. Many single-celled organisms are aerobic and often face oxidative stress, in contrast to multicellular organisms, which are better protected, e.g. due to cover of their skin and controlled delivery of molecular oxygen for cellular metabolism. Thus, fRMsr function may be needed by unicellular organisms more than by multicellular organisms. However, once the enzyme is lost, the ability for methionine sulfoxide reduction might be difficult to replace. Although the importance of fRMsr may have been reduced in multicellular organisms compared to the unicellular, its restoration may enhance stress resistance and support lifespan extension in at least some species.

fRMsr is a relatively new addition to the group of eukaryotic Msrs, whose other members are MsrA and MsrB. MsrA is an oxidoreductase that reduces both protein-based and free Met-*R*-SO and supports three major biological functions: oxidative stress resistance, protein repair, and regulation of protein function. This enzyme has been widely implicated in lifespan control. MsrB has the same protein repair and regulation functions^12^, but does not affect lifespan in *Drosophila*^23^. To explain this observation, one needs to focus on the differences between MsrA and MsrB. One difference is stereospecificity: Met-*S*-SO is a MsrA substrate, and Met-*R*-SO is a MsrB substrate; the other is the unequal activity of these enzymes with free Met sulfoxides. With respect to stereospecificity, the non-enzymatic oxidation of Met by ROS leads to an approximately equal mixture of Met-*S*-SO and Met-*R*-SO, and there is no evidence an isomerase is present that interconverts Met-*S*-SO and Met-*R*-SO. There is an interesting exception, Mical, a stereoselective monooxygenase that converts two conserved Met residues in actin to Met-*R*-SO^34^. However, this function points to an importance of MsrB in regulating protein function. Therefore, the distinct effects of MsrA and MsrB on lifespan control could not be explained by the biased formation of Met-*S*-SO or Met-*R*-SO.

With respect to differences in catalytic efficiency for free Met sulfoxide reduction, we need to first consider the contribution of protein-based Met sulfoxide reduction to lifespan extension. Dabsylated Met sulfoxide and N-acetylmethionine sulfoxide are generally used as substrates for *in vitro* assays of MsrA and MsrB^41^. However, the actual substrates inside the cell are protein-based Met sulfoxides, and their reduction by MsrA and MsrB depends on the accessibility of specific Met residues. Aside from the effectiveness of MsrA and MsrB for the reduction of protein-based Met sulfoxides, one clear fact is that MsrB is very inefficient for the reduction of free Met-*R*-SO, whereas MsrA is highly active with both free and protein-based Met-*S*-SO. As a result, MsrA often exerts a stronger antioxidant protection.

Our findings with fRMsr transgenic flies are consistent with these differences between MsrA and MsrB and suggest that restoration of the ability to reduce free Met-*R*-SO may have beneficial consequences on organismal physiology. The findings also point to the possibility of a combined nutritional and genetic strategy to increase lifespan, wherein a lost function, restored genetically, is supplemented with the substrate used to support this function. In addition, high Met can be toxic, and fRMsr may be viewed as an enzyme that decreases Met toxicity by repairing its oxidatively damaged form. It would be interesting to test additional functions that are lost during evolution of animals and that can be restored using a combination of genetic and nutritional approaches as well as to examine whether combinations of such functions exhibit additive effects on lifespan and stress resistance.

## Electronic supplementary material

Supplementary Information

## References

[1] Sohal RS, Weindruch R (1996). Oxidative stress, caloric restriction, and aging. Science.

[2] Barja G (2013). Updating the mitochondrial free radical theory of aging: an integrated view, key aspects, and confounding concepts. Antioxid Redox Signal.

[3] Vitale G, Salvioli S, Franceschi C (2013). Oxidative stress and the ageing endocrine system. Nat Rev Endocrinol.

[4] Muller FL, Lustgarten MS, Jang Y, Richardson A, Van Remmen H (2007). Trends in oxidative aging theories. Free Radic Biol Med.

[5] Genova ML, Lenaz G (2015). The Interplay Between Respiratory Supercomplexes and ROS in Aging. Antioxid Redox Signal.

[6] Chaiyen P, Fraaije MW, Mattevi A (2012). The enigmatic reaction of flavins with oxygen. Trends Biochem Sci.

[7] Brewer TF, Garcia FJ, Onak CS, Carroll KS, Chang CJ (2015). Chemical approaches to discovery and study of sources and targets of hydrogen peroxide redox signaling through NADPH oxidase proteins. Annu Rev Biochem.

[8] Schieber M, Chandel NS (2014). ROS function in redox signaling and oxidative stress. Curr Biol.

[9] Lu J, Holmgren A (2014). The thioredoxin antioxidant system. Free Radic Biol Med.

[10] Kim HY (2013). The methionine sulfoxide reduction system: selenium utilization and methionine sulfoxide reductase enzymes and their functions. Antioxid Redox Signal.

[11] Boschi-Muller S, Gand A, Branlant G (2008). The methionine sulfoxide reductases: Catalysis and substrate specificities. Arch Biochem Biophys.

[12] Boschi-Muller S, Olry A, Antoine M, Branlant G (2005). The enzymology and biochemistry of methionine sulfoxide reductases. Biochim Biophys Acta.

[13] Lin Z (2007). Free methionine-(R)-sulfoxide reductase from Escherichia coli reveals a new GAF domain function. Proc Natl Acad Sci USA.

[14] Le DT (2009). Functional analysis of free methionine-R-sulfoxide reductase from Saccharomyces cerevisiae. J Biol Chem.

[15] Lee BC, Le DT, Gladyshev VN (2008). Mammals reduce methionine-S-sulfoxide with MsrA and are unable to reduce methionine-R-sulfoxide, and this function can be restored with a yeast reductase. J Biol Chem.

[16] Koc A, Gasch AP, Rutherford JC, Kim HY, Gladyshev VN (2004). Methionine sulfoxide reductase regulation of yeast lifespan reveals reactive oxygen species-dependent and -independent components of aging. Proc Natl Acad Sci USA.

[17] Ruan H (2002). High-quality life extension by the enzyme peptide methionine sulfoxide reductase. Proc Natl Acad Sci USA.

[18] Chatelain E (2013). Evidence for participation of the methionine sulfoxide reductase repair system in plant seed longevity. Proc Natl Acad Sci USA.

[19] Chung H (2010). The Drosophila homolog of methionine sulfoxide reductase A extends lifespan and increases nuclear localization of FOXO. FEBS Lett.

[20] Moskovitz J (2001). Methionine sulfoxide reductase (MsrA) is a regulator of antioxidant defense and lifespan in mammals. Proc Natl Acad Sci USA.

[21] Salmon AB (2009). Lack of methionine sulfoxide reductase A in mice increases sensitivity to oxidative stress but does not diminish life span. FASEB J.

[22] Salmon AB (2016). Effects of transgenic methionine sulfoxide reductase A (MsrA) expression on lifespan and age-dependent changes in metabolic function in mice. Redox Biol.

[23] Shchedrina VA (2009). Overexpression of methionine-R-sulfoxide reductases has no influence on fruit fly aging. Mech Ageing Dev.

[24] Robertson LK, Dey BK, Campos AR, Mahaffey JW (2002). Expression of the drosophila gene disconnected using the UAS/GAL4 system. Genesis.

[25] Shaposhnikov M, Proshkina E, Shilova L, Zhavoronkov A, Moskalev A (2015). Lifespan and Stress Resistance in Drosophila with Overexpressed DNA Repair Genes. Sci Rep.

[26] Shchedrina VA (2011). Analyses of fruit flies that do not express selenoproteins or express the mouse selenoprotein, methionine sulfoxide reductase B1, reveal a role of selenoproteins in stress resistance. J Biol Chem.

[27] Owusu-Ansah E, Song W, Perrimon N (2013). Muscle mitohormesis promotes longevity via systemic repression of insulin signaling. Cell.

[28] Troen AM (2007). Lifespan modification by glucose and methionine in Drosophila melanogaster fed a chemically defined diet. Age (Dordr).

[29] Lee BC (2014). Methionine restriction extends lifespan of Drosophila melanogaster under conditions of low amino-acid status. Nat Commun.

[30] Kabil H, Partridge L, Harshman LG (2007). Superoxide dismutase activities in long-lived Drosophila melanogaster females: chico1 genotypes and dietary dilution. Biogerontology.

[31] Shukla AK (2014). Heat shock protein-70 (Hsp-70) suppresses paraquat-induced neurodegeneration by inhibiting JNK and caspase-3 activation in Drosophila model of Parkinson’s disease. PLoS One.

[32] Girardot F, Monnier V, Tricoire H (2004). Genome wide analysis of common and specific stress responses in adult drosophila melanogaster. BMC Genomics.

[33] Wong R, Piper MD, Wertheim B, Partridge L (2009). Quantification of food intake in Drosophila. PLoS One.

[34] Lee BC (2013). MsrB1 and MICALs regulate actin assembly and macrophage function via reversible stereoselective methionine oxidation. Mol Cell.

[35] Sharov VS, Ferrington DA, Squier TC, Schoneich C (1999). Diastereoselective reduction of protein-bound methionine sulfoxide by methionine sulfoxide reductase. FEBS Lett.

[36] Kand’ar R, Zakova P, Lotkova H, Kucera O, Cervinkova Z (2007). Determination of reduced and oxidized glutathione in biological samples using liquid chromatography with fluorimetric detection. J Pharm Biomed Anal.

[37] Rebrin I, Bayne AC, Mockett RJ, Orr WC, Sohal RS (2004). Free aminothiols, glutathione redox state and protein mixed disulphides in aging Drosophila melanogaster. Biochem J.

[38] Duffy JB (2002). GAL4 system in Drosophila: a fly geneticist’s Swiss army knife. Genesis.

[39] Kaya A, Lee BC, Gladyshev VN (2015). Regulation of protein function by reversible methionine oxidation and the role of selenoprotein MsrB1. Antioxid Redox Signal.

[40] Vermeulen CJ, Loeschcke V (2007). Longevity and the stress response in Drosophila. Exp Gerontol.

[41] Tarrago L (2010). Plant thioredoxin CDSP32 regenerates 1-cys methionine sulfoxide reductase B activity through the direct reduction of sulfenic acid. J Biol Chem.

