# Supplementary material for: Expression of the methionine sulfoxide reductase lost during evolution extends Drosophila lifespan in a methionine-dependent manner
Source: Sci Rep. 2018 Jan 17;8:1010. doi: 10.1038/s41598-017-15090-5 (PMC5798039; doi:10.1038/s41598-017-15090-5)
Supplement: Supplementary file 1 — Supplementary Information [file 41598_2017_15090_MOESM1_ESM.pdf]

Expression of the methionine sulfoxide reductase lost during evolution extends *Drosophila* lifespan in a methionine-dependent manner

Byung Cheon Lee[1,*], Hae Min Lee[1], Sorah Kim[1], Andrei S. Avanesov[2], Aro Lee[1], Bok-Hwan Chun[1], Gerd Vorbruggen[3] and Vadim N. Gladyshev[2,*]

[1]College of Life Sciences and Biotechnology, Korea University, Seoul, 136-712, South Korea, [2]Division of Genetics, Department of Medicine, Brigham & Women's Hospital and Harvard Medical School, Boston, MA 02115 USA, [3]Abteilung Molekulare Entwicklungsbiologie, Max-Planck-Institut für biophysikalische Chemie, Göttingen, Germany

Supplementary Materials

Supplementary Table 1. Lifespan of virgin female and male flies expressing fRMsr on a regular corn meal diet and test of homogeneity of survivorship.

Comparison	GB	Sex	Lifespan			Log-Rank		Wilcoxon		% increase of mean lifespan from control
			Number of flies	Mean ± s.e.m.	Median	χ2	ρ > χ2	χ2	ρ > χ2	
fRMsr2.1	*da-GAL4>yw*	F	115	61.64 ± 1.42	63	27.54	<.0001*	31.87	<0.0001*	18
	da-GAL4>fRMsr2.1	F	114	72.5 ± 1.35	78					
	yw>fRMsr2.1	F	106	62.49 ± 1.44	66	37.47	<.0001*	31.90	<0.0001*	16
	da-GAL4>fRMsr2.1	F	114	72.5 ± 1.35	78					
fRMsr9.1	*da-GAL4>yw*	F	115	61.64 ± 1.42	63	24.49	<.0001*	30.96	<0.0001*	18
	da-GAL4>fRMsr9.1	F	107	72.53 ± 1.29	75					
	yw>fRMsr9.1	F	113	64.01 ± 1.43	69	24.78	<.0001*	22.88	<0.0001*	13
	da-GAL4>fRMsr9.1	F	107	72.53 ± 1.29	75					
fRMsr2.1	*da-GAL4>yw*	M	108	58.44 ± 1.48	61.5	60.53	<.0001*	58.79	<0.0001*	27
	da-GAL4>fRMsr2.1	M	112	74.06 ± 1.21	78					
	yw>fRMsr2.1	M	113	63.11 ± 1.21	66	31.2	<.0001*	34.59	<0.0001*	17
	da-GAL4>fRMsr2.1	M	112	74.06 ± 1.21	78					
fRMsr9.1	*da-GAL4>yw*	M	108	58.44 ± 1.48	61.5	35.52	<.0001*	30.50	<0.0001*	19
	da-GAL4>fRMsr9.1	M	111	69.81 ± 1.43	75					
	yw>fRMsr9.1	M	111	63.05 ± 1.49	69	17.02	<.0001*	12.04	0.0005*	11
	da-GAL4>fRMsr9.1	M	111	69.81 ± 1.43	75					

GB: Genetic background

*: Statistically significant difference

**: % increase of mean lifespan over flies on the control diets

Supplementary Table 2. Lifespan of mated female and male flies expressing fRMsr on the second type of regular diet and test of homogeneity of survivorship.

Comparison	GB	Sex	Lifespan			Log-Rank		Wilcoxon		% increase of mean lifespan from control
			Number of flies	Mean ± s.e.m.	Median	χ2	ρ > χ2	χ2	ρ > χ2	
fRMsr2.1	*da-GAL4>yw*	F	309	55.30 ± 0.64	57	242.01	<.0001*	200.90	<.0001*	24
	da-GAL4>fRMsr2.1	F	298	68.72 ± 0.65	72					
	yw>fRMsr2.1	F	313	57.75 ± 0.67	60	154.32	<.0001*	138.66	<.0001*	19
	da-GAL4>fRMsr2.1	F	298	68.72 ± 0.65	72					
fRMsr9.1	*da-GAL4>yw*	F	309	55.30 ± 0.64	57	233.59	<.0001*	207.68	<.0001*	24
	da-GAL4>fRMsr9.1	F	309	68.38 ± 0.57	69					
	yw>fRMsr9.1	F	319	59.04 ± 0.54	60	172.35	<.0001*	136.79	<.0001*	16
	da-GAL4>fRMsr9.1	F	309	68.38 ± 0.57	69					
fRMsr2.1	*da-GAL4>yw*	M	317	53.84 ± 0.63	57	218.60	<.0001*	166.69	<.0001*	25
	da-GAL4>fRMsr2.1	M	298	67.12 ± 0.75	69					
	yw>fRMsr2.1	M	321	59.39 ± 0.56	60	119.46	<.0001*	79.77	<.0001*	13
	da-GAL4>fRMsr2.1	M	298	67.12 ± 0.75	69					
fRMsr9.1	*da-GAL4>yw*	M	317	53.84 ± 0.63	57	216.70	<.0001*	170.44	<.0001*	25
	da-GAL4>fRMsr9.1	M	299	67.14 ± 0.72	69					
	yw>fRMsr9.1	M	314	59.72 ± 0.61	60	96.32	<.0001*	67.80	<.0001*	12
	da-GAL4>fRMsr9.1	M	299	67.14 ± 0.72	69					

GB: genetic background

*: Statistically significant difference

**: % increase of mean lifespan over flies on the control diets

Supplementary Table 3. Survival time of male flies expressing fRMsr on a diet containing paraquat without or with 1 mM Met and test of homogeneity of survivorship.

Conditions	GB	Sex	Surviving time (Hours)			Log-Rank		% increase of mean lifespan from control
			Total number	Number in each vial	Mean ± s.e.m.	χ2	ρ > χ2	
Paraquat treated fRMsr2.1 (No Met added)	*da-GAL4>yw*	M	60	15	69.8 ± 3.5	22.84	<.0001*	36
	da-GAL4>fRMsr2.1	M	60	15	94.6 ± 3.7			
	yw>fRMsr2.1	M	60	15	78.2 ± 4.12	7.43	0.0064*	21
	da-GAL4>fRMsr2.1	M	60	15	94.6 ± 3.7			
Paraquat treated fRMsr9.1 (No Met added)	*da-GAL4>yw*	M	60	15	69.8 ± 3.5	3.43	0.064	15
	da-GAL4>fRMsr9.1	M	60	15	80.4 ± 3.28			
	yw>fRMsr9.1	M	60	15	74.0 ±2.88	3.62	0.057	9
	da-GAL4>fRMsr9.1	M	60	15	80.4 ± 3.28			
Paraquat treated fRMsr9.2 (No Met added)	*da-GAL4>yw*	M	60	15	69.8 ± 3.5	9.48	0.0021	21
	da-GAL4>fRMsr4.2	M	60	15	84.2 ± 4.18			
	yw>fRMsr4.2	M	60	15	82.6 ± 3.87	0.71	0.3991	2
	da-GAL4>fRMsr4.2	M	60	15	84.2 ± 4.18			
Paraquat treated fRMsr2.1 (Met added)	*da-GAL4>yw*	M	60	15	59.6 ± 2.95	50.33	<0.0001 *	63
	da-GAL4>fRMsr2.1	M	60	15	97.2 ± 4.02			
	yw>fRMsr2.1	M	60	15	67.0 ± 3.53	34.71	<0.0001 *	45
	da-GAL4>fRMsr2.1	M	60	15	97.2 ± 4.02			
Paraquat treated fRMsr9.1 (Met added)	*da-GAL4>yw*	M	60	15	59.6 ± 2.95	15.42	<0.0001 *	29
	da-GAL4>fRMsr9.1	M	60	15	77.0 ± 3.78			
	yw>fRMsr9.1	M	60	15	63.4 ± 2.75	13.07	0.0003*	21

	da-GAL4>fRMsr9.1	M	60	15	77.0 ± 3.78			
Paraquat treated fRMsr4.2 (Met added)	*da-GAL4>yw*	M	60	15	59.6 ± 2.95	6.79	0.0092	17
	da-GAL4>fRMsr4.2	M	60	15	69.8 ± 4.14			
	yw>fRMsr4.2	M	60	15	69.8 ± 4.26	0.048	0.8273	0
	da-GAL4>fRMsr4.2	M	60	15	69.8 ± 4.14			

GB: genetic background

*: Statistically significant difference

**: % increase of mean lifespan over flies on the control diets

Supplementary Table 4. Lifespan of male flies expressing fRMsr on a chemically defined diet containing 0, 1, 10, or 100 mM Met and test of homogeneity of survivorship.

Conditions	GB	Sex	Lifespan			Log-Rank		Wilcoxon		% increase of mean lifespan from control
			Number of flies	Mean ± s.e.m.	Median	χ2	ρ > χ2	χ2	ρ > χ2	
0mM Met in the defined medium	*da-GAL4>yw*	M	116	37.94 ± 1.22	40.5	0.26	0.6121	0.12	0.7249	1
	da-GAL4>fRMsr2.1	M	111	38.30 ± 1.06	39					
	yw>fRMsr2.1	M	123	37.83 ± 1.01	39	0.07	0.7912	0.07	0.795	1
	da-GAL4>fRMsr2.1	M	111	38.30 ± 1.06	39					
	da-GAL4>yw	M	116	37.94 ± 1.22	40.5	1.56	0.212	1.27	0.26	-4
	da-GAL4>fRMsr9.1	M	113	36.61 ± 1.17	39					
	yw>fRMsr9.1	M	110	36.87 ± 1.00	39	0.67	0.413	0.015	0.9031	-1
	da-GAL4>fRMsr9.1	M	113	36.61 ± 1.17	39					
1mM Met in the defined medium	*da-GAL4>yw*	M	97	59.57 ± 1.27	66	0.013	0.9107	0.67	0.4138	5
	da-GAL4>fRMsr2.1	M	104	62.39 ± 0.91	63					
	yw>fRMsr2.1	M	99	61.76 ± 0.91	63	1.00	0.3166	0.33	0.5637	1
	da-GAL4>fRMsr2.1	M	104	62.39 ± 0.91	63					
	da-GAL4>yw	M	97	59.57 ± 1.27	66	1.51	0.2197	0.76	0.3821	4
	da-GAL4>fRMsr9.1	M	97	62.01 ± 0.98	60					
	yw>fRMsr9.1	M	98	61.32 ± 1.09	60	0.0034	0.9534	0.34	0.562	1
	da-GAL4>fRMsr9.1	M	97	62.01 ± 0.98	60					
	da-GAL4>yw	M	106	53.18 ± 1.11	54	46.56	<0.0001*	53.51	<0.0001*	20

	da-GAL4>fRMsr2.1	M	101	63.92 ± 0.85	66					
10mM Met in the defined medium	*yw>fRMsr2.1*	M	98	59.85 ± 0.89	61.5	12.23	0.0005*	12.76	0.0004*	7
	da-GAL4>fRMsr2.1	M	101	63.92 ± 0.85	66					
	da-GAL4>yw	M	106	53.18 ± 1.11	54	23.007	<0.0001*	15.66	<0.0001*	14
	da-GAL4>fRMsr9.1	M	88	60.55 ± 1.30	63					
	yw>fRMsr9.1	M	102	58.65 ±1.07	60	5.51	0.0189*	1.14	0.0285*	3
	da-GAL4>fRMsr9.1	M	88	60.55 ± 1.30	63					

GB: genetic background

*: Statistically significant difference

**: % increase of mean lifespan over flies on the control diets

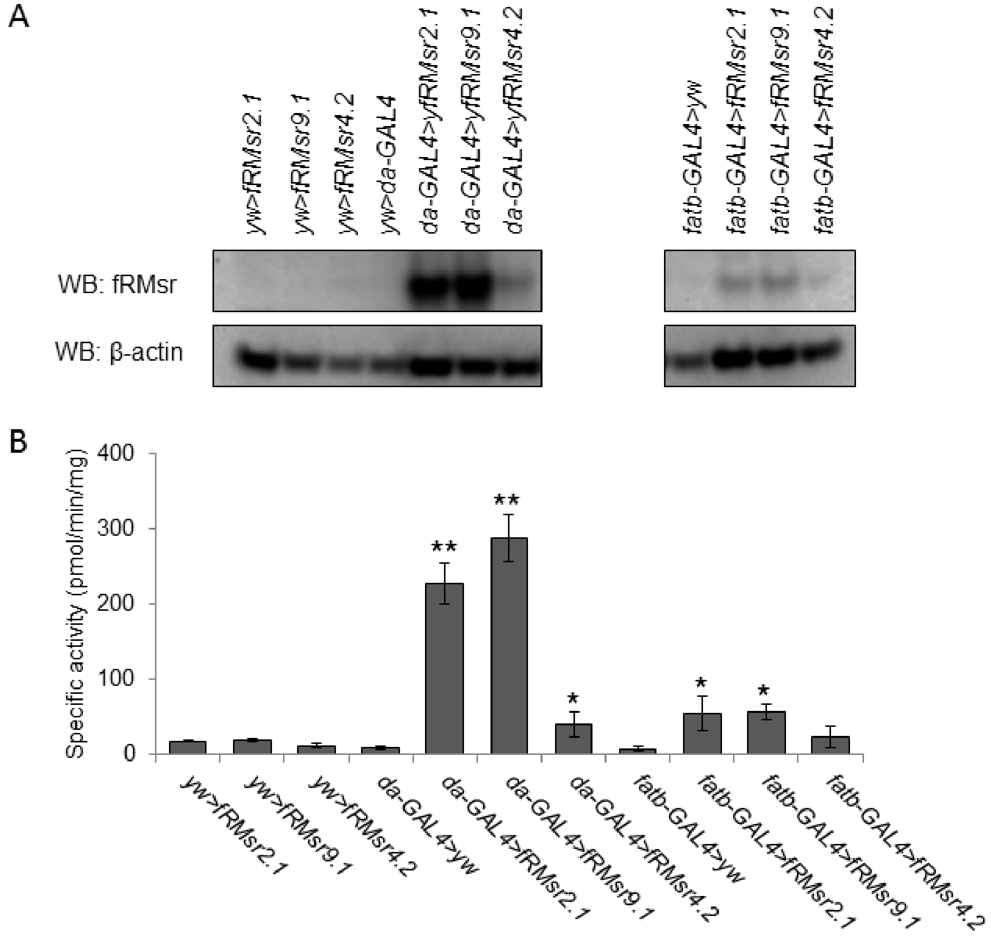

Supplementary Figure 1. Generation of transgenic flies expressing yeast fRMsr in various tissues. (A) Western blotting to examine expression of fRMsr in various tissues and (B) specific activity for free Met-*R*-SO reduction in *da-GAL4>fRMsr2.1*, *da-GAL4>fRMsr9.1*, *da-GAL4>fRMsr4.2*, *da-GAL4>yw*, *fatb-GAL4>fRMsr2.1*, *fatb-GAL4>fRMsr9.1*, *fatb-GAL4>fRMsr4.2*, *yw>fatb-GAL4*, *yw>fRMsr2.1*, *yw>fRMsr9.1*, *yw>fRMsr4.2* (*: $P<0.05$, **: $P<0.01$, Student's *t*-test).

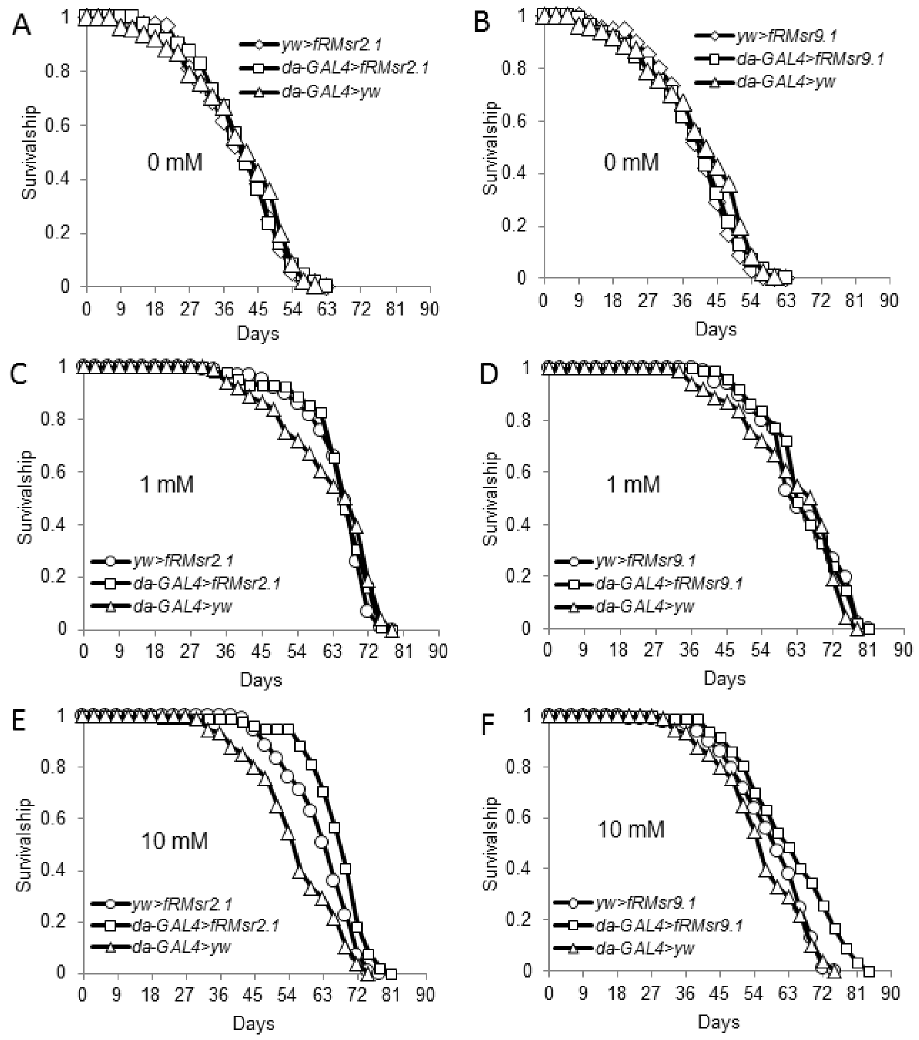

Supplementary Figure 2. Survivorship curve of transgenic male flies expressing fRMsr on the chemically defined diet containing various Met concentration. Survivorship curve of (A) *da-GAL4>fRMsr2.1* and its two controls, *da-GAL4>yw* and *yw>fRMsr2.1*, or (B) *da-GAL4>fRMsr9.1* and its two controls, *yw>da-GAL4* and *yw>fRMsr9.1*, reared on the chemically defined diet containing 0 mM Met concentration. Survivorship curve of (C) *da-GAL4>fRMsr2.1* and its two controls, *da-GAL4>yw* and *yw>fRMsr2.1*, or (D) *da-GAL4>fRMsr9.1* and its two controls, *da-GAL4>yw* and *yw>fRMsr9.1*, reared on the chemically defined diet containing 1 mM Met concentration. Survivorship curve of (E) *da-GAL4>fRMsr2.1* and its two controls, *da-*

GAL4>yw and *yw>fRMsr2.1*, or (F) *da-GAL4>fRMsr9.1* and its two controls, *da-GAL4>yw* and *yw>fRMsr9.1*, reared on the chemically defined diet containing 10 mM Met concentration. See Methods for composition of the chemically defined diet and Supplementary Table 5 for *n* number and statistics of individual strain.

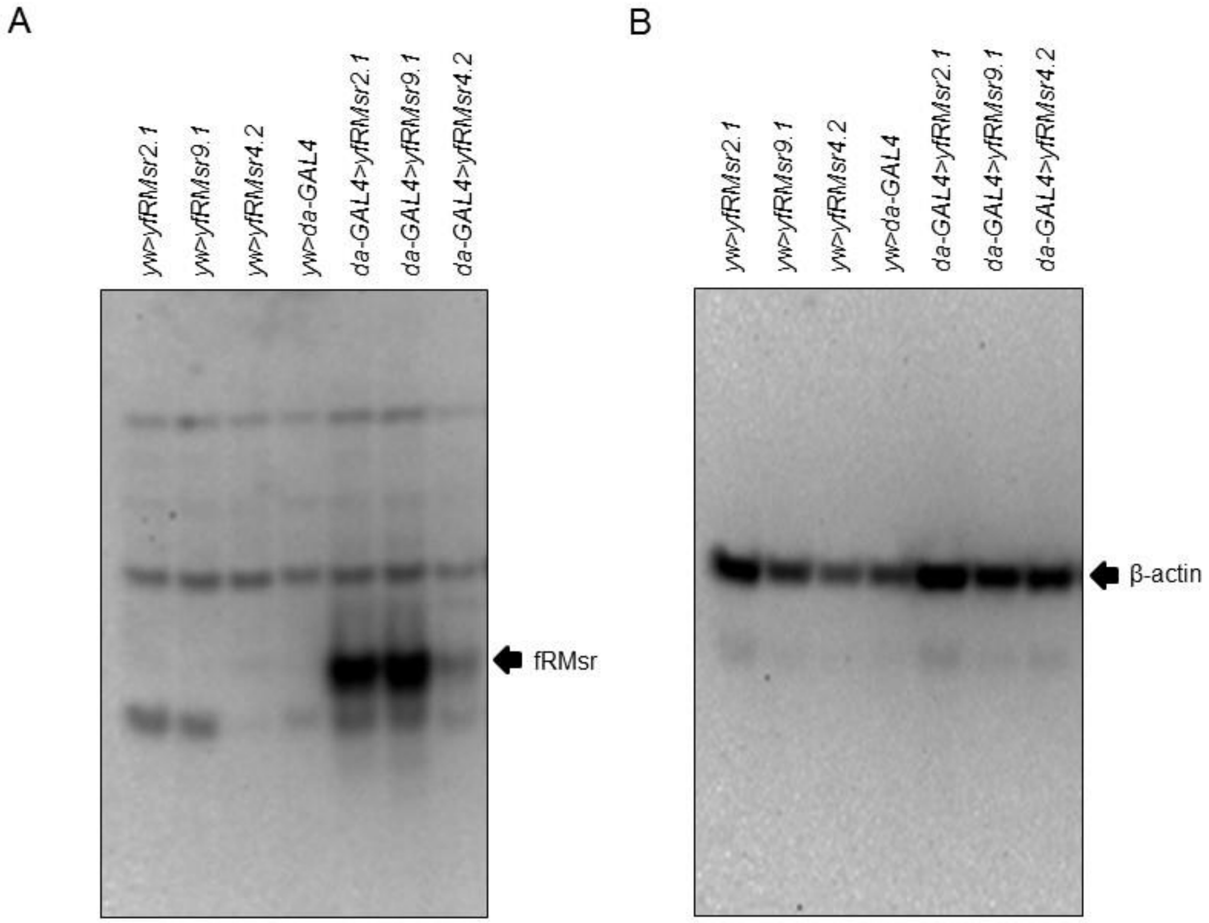

Supplementary Figure 3. Full-length image of western blot analysis. (A, B) All figures are full images of western blots of fRMsr and β-actin in *da-GAL4>fRMsr2.1*, *da-GAL4>fRMsr9.1*, *da-GAL4>fRMsr4.2*, *da-GAL4>yw*, *yw>fRMsr2.1*, *yw>fRMsr9.1*, *yw>fRMsr4.2* lines in Figure 1.
